# Factors Influencing Participation and Engagement in a Teen Safe Driving Intervention: A Qualitative Study

**DOI:** 10.3390/ijerph21070928

**Published:** 2024-07-16

**Authors:** Dominique M. Rose, Cynthia J. Sieck, Archana Kaur, Krista K. Wheeler, Lindsay Sullivan, Jingzhen Yang

**Affiliations:** 1 Center for Injury Research and Policy, Abigail Wexner Research Institute at Nationwide Children’s Hospital, Columbus, OH 43205, USA; dominique.rose@nationwidechildrens.org (D.M.R.); archana.kaur@nationwidechildrens.org (A.K.); krista.wheeler@nationwidechildrens.org (K.K.W.); 2 Center for Health Equity, Dayton Children’s Hospital, Dayton, OH 45404, USA; sieckc@childrensdayton.org; 3 Boonshoft School of Medicine, Wright State University, Dayton, OH 45435, USA; 4 Division of Health Sciences, School of Health and Rehabilitation Sciences, College of Medicine, The Ohio State University, Columbus, OH 43210, USA; lindsay.sullivan@osumc.edu; 5 Department of Pediatrics, College of Medicine, The Ohio State University, Columbus, OH 43210, USA

**Keywords:** parent communication, program implementation, safe driving, teen drivers, qualitative

## Abstract

(1) Background: Few teen driving safety programs focus on increasing parental engagement with high-risk teen drivers, specifically those with a traffic violation. This study explored parents’/guardians’ (‘parents’) experiences with a teen driving safety program, ProjectDRIVE, including facilitators and barriers to program engagement. (2) Methods: We conducted virtual, semi-structured interviews with parents who completed ProjectDRIVE, which included in-vehicle driving feedback technology and individualized virtual training with parents on effective parent–teen communication. (3) Results: Twenty interviews (with 17 females and three males) were transcribed verbatim and independently coded by three coders using systematic, open, and focused coding. Three major themes were identified: factors influencing a parent’s initial decision to participate, factors influencing continued engagement, and perceived benefits of participation. The decision to participate was influenced by these subthemes: parental motivation to help their teen, perceived program usefulness, program endorsement, program incentives, parents’ busy schedules, and lack of access to a car/internet. Subthemes impacting continued engagement included enhanced communication skills, teen willingness to engage, strong parental engagement, and teens’ other priorities. Perceived benefits included greater self-efficacy in communication, improved communication patterns and frequency, and enhanced parent–teen relationships. (4) Conclusions: These findings may set the foundation for developing and implementing future court-ordered parent-based teen safe driving programs for teens with traffic citations.

## 1. Introduction

Drivers ages 16 to 17 continue to have the highest crash rates of all drivers, leading to high rates of injury for themselves, their passengers, and others involved [1,2,3]. Teenage crash risk is particularly high during the first six months of unsupervised driving [4,5,6]. Per mile driven, teen drivers ages 16–19 have a fatal crash rate almost three times higher than drivers ages 20 and older [2,3]. Teen drivers ages 16–17 who have committed a traffic violation have an even greater risk than their counterparts [7,8,9].

Parents are pivotal in educating, supervising, and reinforcing safe driving behaviors of their teens [10,11,12,13,14,15,16]. In addition to controlling access to the car, parents strongly influence their teens’ driving skills and safe driving behaviors, as well as holding their teens accountable for abiding by driving rules that keep them safer on the road [10,13,16]. However, parent’s direct supervision is only mandated during the learner phase; when teens begin to drive unsupervised, parental involvement in their teen’s driving is greatly reduced, even though teens are continuing to gain driving experience and improve their skills [1,5,10].

Numerous driving-safety interventions have used driving feedback technology to promote parental engagement in teen driving, many of which have demonstrated efficacy in improving teens’ driving safety [17,18,19,20,21,22,23,24]. Nonetheless, most existing interventions target teen drivers during their supervised learner stage [10,16,21,22,24,25,26,27,28]. Few interventions have been implemented after teens begin driving unsupervised [17,20,23], and even fewer have targeted high-risk teen drivers, such as those who have a traffic violation [29,30,31]. This significant research gap highlights the need to develop and test comprehensive, parent-focused teen driving safety programs for teens with traffic violations during the unsupervised phase.

According to Ohio law, a teen driver under 18 years old who is cited for a traffic violation must appear in Juvenile Traffic Court along with a parent to confess or deny that they committed the traffic violation [32]. Following a traffic violation, this court appearance provides an ideal opportunity for parent-based driving safety programs. ProjectDRIVE, a parent-based, randomized control trial, recruited and enrolled teen drivers and a parent in a driving safety program that aims to improve parent–teen communication about safe driving practices, and ultimately increase safe driving behaviors and reduce traffic violation recidivism among teen drivers cited for a traffic violation [33]. ProjectDRIVE includes two intervention components: (1) an in-vehicle driving feedback device and an app that provides direct feedback to teen drivers and bi-weekly summary reports to the teens and parents and (2) individualized virtual parent training sessions that focus on effective strategies utilizing motivational interviewing (MI) principles to communicate with teens about driving safety, along with an online communication guide. A total of 240 parent–teen dyads are enrolled and randomly assigned into one of three groups using stratified block randomization: (1) control, (2) driving feedback only, or (3) driving feedback plus parent training. Participating dyads are followed for six months with three months of active intervention. The detailed descriptions of the study protocol of ProjectDRIVE are reported elsewhere [33].

This qualitative study aimed to explore parents’/guardians’ (‘parents’) experiences participating in ProjectDRIVE, including facilitators and barriers to program participation and engagement. Understanding parents’ perspectives and experiences may help identify strategies to successfully deliver court-ordered teen safe driving programs that actively engage parents following their teen’s traffic violation.

## 2. Materials and Methods

### 2.1. Study Design and Participants

Using a qualitative approach, we interviewed 20 parents who completed ProjectDRIVE. To be eligible, parents needed to meet the following inclusion criteria: have a teen driver aged 16 or 17 with a moving-related traffic citation, present to a Juvenile Traffic Court to process their teen’s citation, and complete, along with their teen, the two components of the ProjectDrive intervention. At the time of completing ProjectDRIVE, eligible parents were invited by research staff to participate in an interview exploring their experiences. If interested, a virtual interview was scheduled. The Institutional Review Board approved this study at the authors’ institution (IRB17-00318 [NIH]). This report follows the Standards for Reporting Qualitative Research reporting guidelines for qualitative research [34].

### 2.2. Interview Guide

A semi-structured, in-depth interview guide was developed. The RE-AIM evaluation framework informed the interview guide [35], as well as the Consolidated Framework for Implementation Research (CFIR) [36] and literature on implementation science [37]. The interview guide was pilot-tested and revised based on feedback from experts outside the research team (i.e., a qualitative researcher and a public health researcher with expertise in teen driving safety) before it was finalized. For this study, we analyzed interview questions asking about parent’s motivation to participate and complete ProjectDRIVE activities (e.g., “Can you please tell me a little bit about what motivated you to decide to participate in the program?”) along with perceived barriers (e.g., “What barriers might prevent a parent from participating in the program?”) and facilitators to participation (e.g., “What factors contributed to program participation?”). We also analyzed the questions about parents’ perspectives on the factors influencing their teen’s participation and engagement in ProjectDRIVE. Finally, we analyzed the questions about parents’ views about the potential for juvenile traffic courts to adopt ProjectDRIVE as part of their routine practices.

### 2.3. Data Collection

Twenty virtual interviews were conducted by two trained interviewers (D.M.R., A.K.) and recorded via Microsoft Teams^®^ between November 2021 and December 2023. Interviews lasted approximately 60 min (range = 45–70 min). Participants provided electronic consent before the interview. All recorded interviews were sent to a professional transcription company for verbatim transcription after each interview, with each participant given a unique identification number to protect their identity. The transcriptions were verified by a research team member (DMR) and then uploaded to ATLAS.ti™ (Version 22) [38], a qualitative data management software, to facilitate the coding and analysis process.

### 2.4. Data Analysis

The transcribed data were analyzed using a thematic approach [39,40]. A coding template (i.e., initial codebook) was created based on the interview guide, defining broad categories of findings to enable coding of the interview responses, along with all operational definitions and inclusion/exclusion criteria. Research staff (D.M.R.) applied the initial codebook to the three transcripts to confirm that coding definitions were meaningful and then began to identify new codes. To ensure accuracy and consistency in coding (i.e., intercoder reliability), two additional research team members (A.K. and T.S.) independently double-coded the three interview transcripts using the initial codebook to ensure consistency and to review the identified codes before refining and finalizing the codebook. Intercoder reliability was established, and frequent meetings were held between research team members (D.M.R., A.K., T.S., and J.Y.) to ensure agreement and resolve discrepancies. Once the codebook was finalized, the three coders (D.M.R., A.K., T.S.) engaged in focused coding of the 20 interview transcripts using ATLAS.ti™ software [38]. At least two team members coded each transcript. The research team met regularly to review new codes, discuss conflicts in data interpretation between coders, and resolve discrepancies with group consensus. The coders then coded the transcripts using the agreed-upon themes before synthesizing data into meaningful themes and subthemes [41]. This process was repeated until no new themes were identified.

## 3. Results

### 3.1. Participant Demograpic Introfornation

Of the 20 parents interviewed, most were female (*n* = 17, 85%), White (*n* = 17, 85%), and had a 4-year college graduate degree or higher (*n* = 16, 80%) (Table 1). The average age of participants was 49 years (standard deviation [SD] = 5.9), and half of the parents were female with a male teen driver (*n* = 10, 50%). About two-thirds of parents (*n* = 13, 65%) reported that the teen who received a traffic violation was the first child in their household they taught to drive, and 85% (*n* = 17) reported a household income of $80,000 or greater. Among the teens, speeding was the most frequent traffic citation (*n* = 8, 40%), followed by failure to yield (*n* = 6, 30%).

### 3.2. Major Themes

Three major themes were identified from the parent interviews: (1) the decision to participate in ProjectDRIVE, (2) continued engagement in ProjectDRIVE, and (3) the perceived benefits of participating in ProjectDRIVE (Table 2).

#### 3.2.1. Theme 1: Decision to Participate in ProjectDRIVE

The decision to participate in ProjectDRIVE was operationalized as factors facilitating or impeding the parent–teen dyad’s decision to participate in the study because one of the eligibility criteria was that both the parent and teen agreed to participate [33]. We identified six subthemes, including four facilitators (parent motivation to help their teen, parent perceived usefulness of ProjectDRIVE, program endorsement, and program incentives) and two potential barriers future participants may face to participation in ProjectDRIVE (parent busy schedule and lack of access to a car and/or internet).

##### Facilitators

***Parent motivation to help their teen.*** Most parents noted that the decision to participate in ProjectDRIVE stemmed from a motivation to help their teens drive more safely. Several parents noted that their teens are still learning to drive and thus need more driving practice and feedback to fully develop their driving skills. All parents highlighted how they wanted to help their teen and believed participating in ProjectDRIVE would be a great opportunity to reinforce good driving behaviors and help their teen be a safer and more skilled driver. One parent said, “I think this is a good thing to try to prevent accidents…and just to be more aware of ways to get into a better conversation regarding this stuff” (PT_036, Female). Some parents reported that the potential positive outcomes of the program (i.e., reducing risky driving behaviors among teens) encouraged them to participate, as did the opportunity to reengage in their teen’s driving to promote safer driving practices. One parent commented, “I saw the inherent good that it can do… And my son was going to participate whether he wanted to or not” (PT_049, Female), suggesting parents have an influential role if this was not a research program.

***Parent perceived usefulness of ProjectDRIVE.*** Parents perceived ProjectDRIVE as a useful driver education resource for teens following a traffic citation. In addition to increasing parental involvement in their teen’s driving practices, one participant mentioned, “For me, it was like taking an opportunity to use other resources and opportunities other than me. As a parent, sometimes your kids, are more likely to listen to other people…sometimes they don’t want to hear it all from me” (PT_047, Male).

***Program endorsement.*** Learning about the positive aspects of ProjectDRIVE from other participating parents, research team members, and court staff encouraged some parents to participate in the program. For example, one participant expressed, “I think having the [county leader] be an advocate for [ProjectDRIVE] is great” (PT_124, Female). Another participant noted, “I think hearing from other people is always helpful. ‘Why did you do it? And how did it help you?’ Because then it might help me…” (PT_031, Female).

***Program incentives.*** Parents conveyed that receiving monetary compensation also motivated their initial decision to participate. Several parents noted that compensation for participating in the program was a bonus for both them and their teen, as stated by one parent: “Well, I mean, if you were going to help me teach my son to be a better driver and pay me to do it, then this wasn’t even a question. I was like, ‘Okay, you mean I have to help my son do something better, and you’ll pay me to do it? For sure, absolutely’” (PT_054, Female).

##### Barriers

***Parents’ busy schedules***. Several parents indicated that their busy schedule impacted their initial decision to participate in ProjectDRIVE. One noted, “…The hardest thing was scheduling time…just simply because my life is crazy” (PT_031, Female). Between parental responsibilities and teens’ busy schedules (e.g., sports or extracurricular activities), finding time to complete study activities was a barrier to participation for the interviewed parent and likely also for parents who chose not to participate. The flexibility of ProjectDRIVE and the program schedule was very helpful in overcoming this barrier, as noted by a parent: “I’d say maybe time—A time conflict if a parent is working. For example, I work first shift, so it worked out a little bit better, but if a parent is working second and third shifts and the child is not available at the time, I would say time conflict” (PT_117, Female).

***Lack of access to a car or the internet.*** A few parents mentioned that not having access to a car or the internet could be potential barriers to some families participating in ProjectDRIVE if this intervention were to be implemented as a court-mandated program in the future. Two of the eligibility criteria of the ProjectDRIVE research study were for teens to have access to a vehicle and for them to be the primary drivers of that vehicle. One participant mentioned, “…Some kids don’t have their own car, so that would be kind of be an issue…” (PT_086, Female). Another parent stated, “If the parent doesn’t have access to internet in his or her home… it would be harder for them to focus on this program” (PT_115, Female). Lack of internet connectivity in the home would make it harder for families to use all program components, as the intervention was delivered virtually, and the study website was used to provide examples of MI-style conversation starters for parents.

#### 3.2.2. Theme 2: Continued Engagement in ProjectDRIVE

Continued engagement in ProjectDRIVE was operationalized as facilitators and barriers to the parent–teen dyad’s decision to complete all required program activities. We identified four subthemes, including three facilitators (enhanced communication skills, teen willingness to engage, strong parental engagement) and one barrier (teen’s other priorities).

##### Facilitators

***Enhanced communication skills.*** Enhanced communication skills were operationalized as improved parental ability to effectively communicate about safe driving practices with their teen. Most parents reported not having the necessary skills or confidence to discuss safe driving practices with their teens, although they continued advising their teens about driving. For instance, one parent mentioned, “What I liked most is it spurred me thinking about how to frame questions or conversations rather differently. I think the natural instinct is to just tell kids what to do and how to do it. I liked how asking open-ended questions engag[ed] them in the conversation. They get a lot less defensive when you speak in that way. That was good just to have very specific examples of how you could frame a question and generate a conversation” (PT_092, Female). Several parents reported their prior training in strategies to promote effective parent–child communication. Still, they highlighted how they appreciated the refresher on using these skills to promote safe driving practices in their teens. One participant commented, “I think they’re definitely skills that every parent needs to have, and even if we use them all the time, we need a reminder and a refresher; the conversations with our kids matter just as much as the conversations we’re having with the difficult person at work” (PT_031, Female). One participant explained, “Because of my professional background, that’s something that I have. But it’s always good to have someone to point out ideas of [different] ways you can say things instead of just telling them what they have to do…” (PT_036, Female).

***Teen willingness to engage.*** ProjectDRIVE requires both the parent and the teens’ participation in program activities [33]. The teens’ willingness to engage in the program significantly impacted their parents’ continued engagement with ProjectDRIVE. Some parents reported having difficulty continuing to engage in the program without the teen’s buy-in and commitment. Some parents explained that their teen’s initial reluctance to participate in the program was a barrier to program participation. One parent explained, “She definitely rolled her eyes. She was like, ‘Why do you need me to sign up for this?’ She did question why, but whenever [she did], I said, ‘This is because there are consequences for your actions, and we want you to be safe…’ So, once we got started, she was fairly positive about it” (PT_080, Female). Another parent stated, “So anytime one of my kids expresses interest in doing something, I feel like it’s going to be helpful to them. I’m all in whatever it is, let’s go” (PT_031, Female).

***Strong parental engagement.*** Parental engagement was operationalized as engaging in safe driving conversations before program participation. Strong parental engagement before program participation positively influenced engagement in ProjectDRIVE and communication with their teen about safe driving practices. One participant explained, “So, we have defined that from the beginning with our children, and even had them write out something, kind of a contract between the parent and child that just gives them a good understanding that will be the expectation” (PT_012, Female). Another participant explained, “I would say parents [having a] good attitude about [their teens driving safe] and not making it a drudgery [thing] to do. I think that with me being positive…her reports were mostly positive…So I was like, ‘It does look like you’re doing a good thing’… And I think that made her more willing to persevere on…if parents are positive towards the results and use that questioning [open-ended questions, affirmations, reflective listening, and summarizing statements (OARS) communication strategies] whenever the results aren’t as good…” (PT_080, Female).

##### Barrier

***Teen’s other priorities.*** As previously mentioned, parents explained that their teen’s busy schedules were often a barrier to participating in the program, particularly the parent–teen communication component. One participant commented, “[There] might be sometimes [when I’m] working and when kids have their own activities, that can be kind of difficult (PT_036, Female).

#### 3.2.3. Theme 3: Perceived Benefits of Participating in ProjectDRIVE

The final theme identified was the perceived benefits of participating in ProjectDRIVE. This theme was operationalized based on the parents’ views of the program’s benefits. Three subthemes were identified (greater self-efficacy in communication, improved communication patterns and frequency, and enhanced parent–teen relationship), all of which were facilitators of participating in ProjectDRIVE.

##### Facilitators

***Greater self-efficacy in communication.*** Greater self-efficacy in communication was operationalized as improvements in the parents’ confidence in their ability to effectively communicate with their teens about safe driving. One participant mentioned, “I did feel like, in general, I was able to think through what kind of things I was going to talk about and how I would use some of those strategies during a conversation. And it becomes more ingrained in your conversations as you go, I thought, especially by the end” (PT_080, Female). Another participant mentioned, “I think some of that stuff [communication strategies] is not natural for me, so it’s going to be hard to not want to default back to traditional, maybe less positive parenting styles” (PT_092, Female).

***Improved communication patterns and frequency.*** The subtheme of improved communication patterns and frequency was defined as increased quantity and quality of conversations about safe driving topics with their teen [11]. One participant noted, “Why not take advantage of the opportunity to participate to get some of our own feedback? As far as communication, it [has] certainly set up an environment where it forced us to talk about some things. Not only did the program itself encourage us to do that, but there was some sort of organic or natural discussion that took place over the course of the study that were opportunities to talk about how her driving was doing and some opportunities for me to ask some questions” (PT_047, Male). Some participants highlighted how this program prompted conversations with their teens about safe driving behaviors and emphasized the importance of reinforcing safe driving practices. For example, one parent explained, “I feel like I’m still doing that with him. We live in the country, and we’re a highway-type road out front, and we have fog, and you could slide into the path of somebody with the ice and things like that. So as the weather was changing, it was a positive thing, just a reminder [to have safe driving conversations]” (PT_071, Female).

***Enhanced parent–teen relationship.*** The enhanced parent–teen relationship was operationalized as improving the parent–teen relationship due to increased communication following their teen’s traffic citation/violation. Some participants reported that ProjectDRIVE improved their parent–teen communication, which improved their relationship with their child following the traffic citation. Such improved parent–child communication and relationships could benefit them in the long run. For instance, one participant stated, “So, helping parents learn how to have conversations with their teens is such a benefit for their relationships for the rest of their lives on beyond driving and safety and any of those things, it’s something that everybody needs an opportunity to learn” (PT_031, Female).

## 4. Discussion

This study explored parents’ experiences with ProjectDRIVE, identifying the facilitators and barriers to participating in and engaging with ProjectDRIVE. Our results highlight the importance of parents’ intrinsic motivation to participate in teen driving safety programs. The findings also showed that the period following a traffic violation may provide a “window of opportunity” to engage parents in a safe teen driving program. In addition to parent commitment, the teen’s willingness to engage in the project played an important role in the dyad’s continued engagement in ProjectDRIVE. Meanwhile, busy parent schedules and other priorities for teens acted as barriers to participation and/or engagement in ProjectDRIVE. Parents highlighted several benefits from participation, including increased self-efficacy in communication with their teens, improved communication style and frequency, and enhanced relationships. Our findings have important implications for implementing parent-based teen driving safety interventions among high-risk teen drivers with a traffic violation [6,8,30]. Parent-based teen driving safety programs should use a traffic citation as a valuable “teachable moment” to empower parents with effective communication skills to motivate their teens to be safer and more responsible drivers [31,42,43].

When a teen receives a traffic citation, parents may be willing to take proactive steps to address the situation, but they may not feel well-prepared to do so [10,11,15]. Parents noted that their decision to participate in ProjectDRIVE was facilitated by their motivation to help their teen be a responsible driver, with many parents highlighting how they perceived ProjectDRIVE as a useful resource to help their teen following a traffic citation. However, very few programs are available to parents of teens with traffic violations despite their heightened risk for re-offense, crashes, and crash-related injuries [31,43,44]. Although parents are required to supervise their teens when they are learning to drive [27,45,46], many were not specifically trained in effective communication, such as using MI principles. By using the traffic citation as an opportunity to reinforce safe driving practices, parents can initiate an open and non-judgmental conversation with their teen about the consequences of unsafe driving behaviors and encourage the teen to share their perspective on the citation; this positive reinforcement may help encourage teen drivers to make safer choices on the road [21,47,48].

We identified several facilitators and barriers to participating in ProjectDRIVE that can inform future parent–teen driving interventions. Parents noted their busy schedules could present challenges to participating in teen safe driving interventions. Thus, finding effective ways to address this barrier is crucial for overall program success. Parents indicated that hearing positive aspects of ProjectDRIVE from other participants and court staff and receiving monetary compensation could encourage parents to engage in this program. Working around parents’ schedules and using parent-preferred communication method(s) could aid parents’ participation and engagement [10,22]. Additional research is warranted to identify strategies to address these identified barriers.

Parents stated that active involvement in ProjectDRIVE required continued parental commitment and their teen’s engagement. Open communication between parents and teens about teens’ needs and priorities could help encourage teen engagement. Numerous driving safety interventions have been developed, including an explicit parental component [10]. Still, most existing interventions have focused on setting rules and restrictions to limit driving in high-risk situations, such as late-night driving and driving with other teen passengers [13], without including training for parents on effective communication strategies. Developing safe driving behaviors is difficult to address solely through driving restrictions, especially when teens drive without supervision [10,13]. Some risk-taking is a normal part of adolescent development. Teenagers driving unsupervised could choose unsafe driving behaviors, such as speeding or using a cell phone, to rebel against restrictions [48,49]. ProjectDRIVE trains parents on MI principles to improve and emphasize effective parental communication to motivate teens to make safe driving behavior choices. The emphasis on parent–teen effective communication in ProjectDRIVE can help empower teens to make safe driving behavior choices behind the wheel [11,15,50].

MI principles can be useful tools for parents to engage in effective conversations with their teens about driving safety through fostering open communication and building trust. MI principles have been used to improve parent–child communication in the context of teens’ other safe behavior choices, including healthy eating and condom use [51,52]. Several parents described that the MI principles they learned through ProjectDRIVE included using OARS or open-ended questions, affirmations, reflective listening, and summarizing, encouraging parents to actively listen and motivate behavior change with their teens [53]. Notably, parents explained that the program increased their confidence in communicating with their teens about driving safety, which increased the quality and quantity of conversations they had about safe driving topics. Some parents noted MI principles learned may extend beyond safe driving conversations, indicating that they may have improved their relationships with their teens after utilizing these principles. Our results support the need for designing and implementing parent-based teen driving safety interventions with a direct parent component to improve communication about teen safe driving practices following a traffic violation [31]. These interventions can reengage parents in their teens’ driving practices to prevent traffic violation recidivism, crashes, and crash-related injuries among these teens.

### Limitations

Our study has several limitations. Despite careful methodology, this study was qualitative, and the questions posed to participants could have been misinterpreted, and/or the analysis could have had bias. Most participating parents were female, White, and from more affluent families due to the program’s inclusion criteria, which required the teen to have access to a car or the internet. The opinions and experiences reported in this study only represent those parents who voluntarily participated in ProjectDRIVE and may not reflect the experiences of parents who are male, non-White, from less affluent families, or those who chose not to participate in our study. We also asked parents about their perceptions of their teen’s experience in the program; thus, some of these results may not reflect the actual experiences of teens themselves. Future studies should be conducted with a larger, more diverse sample of parents of teens with a traffic violation, as well as collecting qualitative data with teen drivers themselves.

## 5. Conclusions

This study used a qualitative approach to understand parents’ experiences participating in ProjectDRIVE. The main barriers included parents’ busy schedules and other teen priorities. In contrast, noted facilitators included parents’ motivation to help their teen, the perceived usefulness of the program, and the benefit of improved parent–child communication and relationships. Our results contribute to the literature on potential effective parental engagement strategies to promote safe driving practices among high-risk teens, specifically teen drivers cited for a traffic violation. These findings may inform future strategies to address the barriers to participating in safe driving interventions. These findings may also set the foundation for developing and implementing future court-ordered parent-based teen safe driving programs for teens with a traffic citation.

## Figures and Tables

**Table 1:** Demographic and participant characteristics.

Participant (PT) Number	Parent				Teen
	Parent–Teen Dyads ^1^	Age	Education Level ^2^	Number of Teens Taught to Drive	Citation
PT_038	F-F	53	5	1	Failure to Control
PT_080	F-F	43	5	2	Failure to Control
PT_036	F-F	50	5	2	Failure to Yield
PT_069	F-F	52	5	1	Failure to Yield
PT_117	F-F	42	5	1	Failure to Stop at a Stop Sign
PT_012	F-F	48	4	3	Speeding
PT_145	F-F	46	3	1	Speeding
PT_131	F-M	55	3	3	Failure to Control
PT_031	F-M	56	5	2	Failure to Yield
PT_054	F-M	43	4	1	Failure to Yield
PT_086	F-M	49	4	1	Failure to Yield
PT_099	F-M	51	4	1	Failure to Keep a Safe Following Distance
PT_001	F-M	36	3	1	Speeding
PT_071	F-M	53	5	1	Speeding
PT_092	F-M	47	4	1	Speeding
PT_113	F-M	44	3	1	Speeding
PT_124	F-M	56	4	1	Speeding
PT_049	M-M	56	5	2	Failure to Yield
PT_105	M-M	43	4	1	Speeding
PT_047	M-F	58	5	2	Failure to Obey a Traffic Control Device

^1^ Parent–teen dyad: F = female; M = male. ^2^ Education level: 1 = some high school; 2 = high school graduate; 3 = some college; 4 = four-year college graduate; 5 = some graduate school or higher.

**Table 2:** Themes, subthemes, and sample quotes related to parent’s participation and engagement in ProjectDRIVE.

Theme 1. Initial decision to participate	
Facilitators	
Parent motivation to help their teen	“I decided to participate because…I think this is a good thing to try to prevent accidents…and just to be more aware of ways to get into a better conversation regarding this stuff” [increasing parent–teen communication about safe driving behaviors] (PT_036, Female)
	“We’re in the middle of a pandemic, but we also need to be safe drivers… there’s a lot of people that die every year on the roads, and I just think that I’m glad that we’re focusing on something like this, because I think it’s really crucial. So, I think that’s what compelled us to participate” (PT_012, Female)
Parent’s perceived usefulness of ProjectDRIVE	“Honestly just the way you kind of shared with us how the teenagers [are] going to receive feedback on her driving, and not even just for our personal family and what I felt, like it might help with my teen drivers…and I just felt like this was an excellent way to do that…” (PT_012, Female)
	“…their drivers ed[ucation], it doesn’t make up for experience and feedback that you get. This program is taking place once they’re actually in that stage of getting that experience. I mean, for me, that’s the motivation” (PT_047, Male)
Program endorsement	“The [county leader] that we had truly seemed to care about teens, and he did not want the percentage of teen deaths in his county. That’s, I think, how you get [parents and teens] to buy in; you speak about the kids and the lives that it could save, and you hit the personal side with the [county leader]” (PT_092, Female)
	“I think…word of mouth people saying, ‘Hey, I used this, and I really liked this program, and this is why I like this program, and this is why I found it valuable’” (PT_054, Female)
	“I think probably like having some testimonials that you could share with that traffic court system in another county, you know, maybe parent and child, but also even having your judge provide some sort of a testimonial” (PT_012, Female)
Program Incentives	“The money part was really good. Because she’s working and she’s trying to save money because she knows that with that, she can buy what she wants. So that was a good motivation for her” (PT_036, Female)
	“I’m thankful the program was kind enough to give us some funds. I think the money kept him engaged…so that kept him going” (PT_105, Female)
Barriers	
Parent schedule	“…Biggest barrier to entry, so to speak, is just having a set time where you are sitting down with your teenager…” (PT_012, Female)
	“…People are just so busy that they don’t want to be bothered with one more thing to do, honestly” (PT_086, Female)
	“Sometimes as a parent, since you are coming and going, running work, kids, dog, whatever. Sometimes, it’s hard to keep up with things. I mean, I know I have to record a talk with my daughter, but I just forgot. I mean, I knew I had it…but then life happens. And I get the reminder now like, ‘Okay, now that when we drive to the grocery store, that’s when you’re going to talk. Or we’re watching TV. Okay, I have you here right now. So let’s talk about this’. So it’s kind of hard” (PT_036, Female)
Lack of access to a car/or the internet	“I did it on the computer… So, I don’t know how the setup looks on the phone. I think you would need some kind of internet access and computer access. You could probably do it on the phone, too. But I think you need a certain level of technology to do it. Like if I had to go to the library and do it, I probably wouldn’t have bothered” (PT_069, Female)
	“I feel like some kids don’t have their own car, so that would kind of be an issue…” (PT_086, Female)
Theme 2. Continued engagement in ProjectDRIVE	
Facilitators	
Enhanced communication skills	“It gets the parent more involved in the training outside of nagging at the teen driver while they’ve got their permit, and you’re like, ‘Don’t do this. Don’t do that. Watch this’. Then you can actually structure your conversations a little better” (PT_099, Female)
	“Just think reminding myself of how to have those conversations. How to give him affirmations and not just come directly at him like he’s in trouble or I’m yelling at him. Just trying to have those real conversations where we both don’t feel attacked” (PT_113, Female)
	“I think those things would very much be useful for parents that maybe aren’t comfortable in talking or maybe don’t have those kinds of tools. Those sorts of things I kind of do at work anyway. And I definitely do them with my daughter more so than I did my boys. So, for me, it was more of just reiterating stuff that I already knew. But it was still good stuff to look at again” (PT_099, Female)
Teen’s willingness to engage	“I think the biggest one would be the teen’s willingness to do it. I mean, unless, for some reason, the parent just again doesn’t seem to see any benefit to them…it’s all about the whole ‘what’s in it for me’, right? What do I get out of this?” (PT_031, Female)
	“She knew it was her responsibility to kind of move through that citation and the consequences that came with that, which is why she ended up deciding to go to court…” (PT_012, Female)
	“Well, think[ing] back, [my teen] was interested. So anytime one of my kids expresses interest in doing something, [and] I feel like it’s going to be helpful to them. I’m all in whatever it is, let’s go” (PT_031, Female)
Strong parental engagement	“I think we have just raised our children to understand what a huge responsibility that (driving) is. So when it came time for them to get their license, they already had a pretty good awareness of what a big responsibility it is to have a vehicle on the road with other people. And so they’re very willing to listen to, you know, our guidelines and our feedback with regards to driving” (PT_ 012, Female)
	“My first response is, ‘You better knock it off or give me the keys’. That’s my quick 10-s stuff. That’s effective. He doesn’t like it, and he’ll skirt around it. But if you bring them in and make them more part of the conversation and the choice and discuss options versus just…” (PT_049, Male)
Barriers	
Teen’s other priorities	“…Having a teenager that’s gone all the time…[and] finding the time to really sit down have [safe driving] conversations…are a little bit more tricky because you have to really plan and be intentional…” (PT_012, Female)
	“I think it was probably not high on his to-do list. So he got them done. But with a little bit of nagging” (PT_113 Female)
Theme 3. Perceived benefits of participation and engagement	
Facilitators	
Greater self-efficacy about communication skills	“I felt better about [communicating with my teen] because I had never really heard of that kind of question [communication style]. I don’t know, it made me more aware of that kind of approach, I guess” (PT_069, Female)
	“Yes, I try to do the affirmations. I’m not that good at that, but I’ll have to practice that one. I’m not really good at summarizing. I was really focused on the open-ended questions…so when I’m having conversations with him about school, I am trying to give him the affirmations. Like, ‘Okay, I understand what you’re saying, and I get that you’re concerned…’ I think it teaches you to be a little more patient and try and listen to what they’re actually saying” (PT_086, Female)
Improved communication patterns and frequency	“I think one of the unforeseen, positive consequences of this experience for him is that he’s more open about talking about his driving and talking about, I mean, when we had the horrible snow and ice we had a couple of weeks ago. We had a very long conversation about how you react and what do you do. …” (PT_031, Female)
	“I would say I think asking [my teen] open questions and following up with more questions when she expressed her thoughts helped me realize that I can do that with other things in our lives when it comes to communicating with [my teen]” (PT_117, Female)
	“Just asking her open-ended questions, not just with her driving but also with her life and what’s going on at school, I feel like we’ve always communicated pretty well, but I think it just steps it up a little bit more” (PT_145, Female)
	“But I guess it’s always a good reinforcement of using open-ended questions versus ‘yes’, ‘no’ questions and answers. It was a good reinforcement for what I hope, or I think, we’re already doing” (PT_124, Female)
Enhanced parent–teen relationship	“I think using that technique, it did give me the chance to gather her ideals, her thoughts about driving, or her difficulties when it comes to driving, as well as to speak to her one-on-one as a friend versus a parent/child relationship in a sense. I felt like she was more willing to listen to what I had to say, especially since we were doing the activity. I didn’t get any eye rolls or huffing or puffing when I was speaking to her. It made a difference” (PT_117, Female)
	“I think it just makes for us having a better line of communication. So hopefully it’ll help just in every aspect of our relationship being able to just talk to each other” (PT_113, Female).
	“Because they’re [OARS communication statements] effective and [if you’re] stuck in old ways that are hard to break, so you always can improve. You never stop trying to improve your relationship. Easier said than done” (PT_049, Female)

## Data Availability

The datasets presented in this article are not readily available because the data are part of an ongoing study. Request to access the datasets should be directed to Jingzhen Yang at ginger.yang@nationwidechildrens.org.
